# Factors influencing intentions to use Apple Pay: A behavioral perspective

**DOI:** 10.1371/journal.pone.0327122

**Published:** 2025-07-17

**Authors:** Khalid Waleed Abdo, Imdadullah Hidayat-ur-Rehman, Sultan Bader Aljehani, Esam Mohammed Aloufi, Ali Alshehri

**Affiliations:** 1 Department of MIS, Faculty of Business Administration, University of Tabuk, Tabuk, Saudi Arabia; 2 Department of Computer Sciences, Applied College, University of Tabuk, Tabuk, Saudi Arabia; IU Internationale Hochschule GmbH, GERMANY

## Abstract

The study aims to investigate factors influencing Apple Pay adoption, focusing on lifestyle congruence, perceived security, perceived trust, perceived financial control, ease of use, and relative advantage. By applying the Technology Acceptance Model (TAM) and Diffusion of Innovation (DOI) theory, it seeks to understand these factors’ contributions to user perception and adoption, providing insights into user behavior and enhancing the effectiveness of digital payment solutions. A survey approach was employed, combining stratified random sampling and convenience sampling to collect data from major cities in Saudi Arabia, targeting Apple-pay users. The survey was distributed online via social media platforms. Data from 221 valid responses were analyzed using partial least squares structural equation modeling. The study revealed that the model explains 62.2% of the variance in the intention to use Apple Pay. Key findings include significant impacts of perceived financial control, relative advantage, perceived security, perceived trust, and ease of use on the intention to use Apple Pay. Lifestyle congruence and ease of use influenced usage intentions but not perceived relative advantage. These results underscore the importance of security, trust, and ease of use in driving Apple Pay adoption. The multi-group analysis findings indicate that younger users prioritize security and lifestyle congruence, while females value financial control and lifestyle fit, and males emphasize security and trust in their Apple Pay adoption decisions. This study uniquely integrates the DOI theory and TAM to examine Apple Pay adoption, highlighting the critical roles of perceived financial control, security, and trust. It expands traditional TAM by incorporating financial control, emphasizes security’s impact on trust, and introduces lifestyle congruence as a significant adoption driver. This comprehensive framework provides valuable insights for digital payment adoption.

## 1. Introduction

The rapid advancement of financial technology (FinTech) has transformed the global payments landscape, with digital wallets emerging as a dominant alternative to traditional payment methods [1]. Mobile wallets, such as Apple Pay, Google Pay, and Samsung Pay, offer users a secure, convenient, and contactless way to conduct financial transactions [2]. Among these, Apple Pay has gained significant prominence due to its seamless integration within Apple’s ecosystem, offering enhanced security and ease of use [3]. Given the increasing penetration of smartphones and digital payment services, understanding the behavioral factors influencing the adoption of Apple Pay is crucial.

The significance of mobile wallets was further underscored during the COVID-19 pandemic, which accelerated the shift toward contactless payments. Consumers increasingly preferred digital transactions over cash-based methods due to health concerns and convenience [4]. Saudi Arabia, in line with its Vision 2030 Financial Sector Development Program, has emphasized digital transformation by promoting mobile wallet adoption. In 2019, the Saudi Arabian Monetary Authority (SAMA) launched mobile payment services, aiming to increase digital transactions to 70% of total payments by 2030 [5]. Given this digital shift, it is imperative to investigate the factors that influence consumer adoption of Apple Pay in Saudi Arabia and beyond.

Despite the growing adoption of mobile wallets, a significant portion of smartphone users remains hesitant to use Apple Pay. Although it is one of the most widely available digital payment solutions, many users continue to rely on traditional credit cards and cash transactions [4]. Research suggests that security concerns, trust issues, financial control, and ease of use play a critical role in consumer payment choices [6]. However, there is limited empirical research focusing specifically on Apple Pay and the behavioral factors influencing its adoption. Addressing this gap is essential for understanding consumer reluctance and identifying strategies to enhance mobile wallet adoption.

While various studies have explored digital payment systems, many have examined mobile wallets collectively rather than focusing on specific platforms. Apple Pay, unlike its competitors, operates within a closed ecosystem, which may impact consumer perceptions differently compared to open-platform wallets like Google Pay [5]. Additionally, although previous research has acknowledged the role of security, trust, and convenience in mobile payment adoption, few studies have systematically analyzed how these factors interact within the Apple Pay ecosystem. This study aims to fill this gap by examining the behavioral drivers that influence consumer intention to use Apple Pay.

Another critical gap lies in the geographical context. Existing literature on mobile wallet adoption has largely focused on Western markets, leaving an insufficient understanding of consumer behavior in regions like Saudi Arabia. Given the country’s strategic push toward digitalization and the increasing use of Apple Pay among Saudi consumers, it is crucial to investigate the behavioral factors affecting its adoption in this specific market [2].

The decision to focus on Apple Pay, as opposed to other mobile wallets, is based on several factors. Firstly, Apple Pay holds a dominant position in the mobile payment industry, particularly in premium smartphone segments. It offers a higher level of security than many other digital payment methods by employing tokenization and biometric authentication [4]. Unlike Google Pay, which is available across different operating systems, Apple Pay is exclusively designed for Apple devices, leading to a more integrated user experience.

Moreover, Apple Pay has seen substantial adoption in Saudi Arabia, becoming one of the most preferred mobile payment solutions in the region. Given Saudi Arabia’s regulatory support for digital transformation, understanding the behavioral aspects of Apple Pay adoption can provide valuable insights for policymakers, financial institutions, and service providers [5].

To contribute both academically and practically, this study extends existing digital payment literature by focusing on Apple Pay, addressing a platform-specific research gap. It also integrates behavioral theories to provide a comprehensive understanding of how security, trust, financial control, and ease of use influence mobile wallet adoption. Additionally, the findings will help financial institutions, policymakers, and technology developers improve mobile payment adoption strategies. By identifying key adoption drivers and barriers, stakeholders can develop targeted interventions to enhance consumer trust and usability, ultimately accelerating the transition to a cashless society.

Building on the discussion above, this study aims to:

1. To identify and analyze the key behavioral factors influencing consumers’ intention to use Apple Pay.
2. To propose a model comprising the identified factors and validate it based on empirical analysis.
3. To provide actionable recommendations for enhancing mobile wallet adoption, particularly within the context of Saudi Arabia’s financial digitalization initiatives.

By investigating these objectives, this study will contribute to the growing body of knowledge on mobile payment adoption and offer valuable recommendations for stakeholders in the Fintech industry.

## 2. Theoretical framework

In reviewing customers’ reliability towards any service, previous studies have concentrated more on quality features. This research focuses on the security and customers’ perception of finance control and trust leading to satisfaction and adoption. Apple pay is depending mainly on the diffusion of innovation theory and Technology-Acceptance model. While the components of the diffusion theory and TAM are similar but focus on different concepts, and in recent times, their complexity has risen. TAM theory was developed by Davis and it has two key components of user attitudes that influence technology adoption, perceived usefulness and ease of use. The relationship between the attitude towards using a system and its actual use is affected by the intent to use it. If a technology improves the user’s performance, it is considered valuable, and the chance will be increased for its adoption by the users. TAM is a suitable model for assessing customer’s behavior in terms of digital payments. The theories of TAM are comparatively simple and make it easy to understand, access, and apply [7,8].

The diffusion of innovation theory is proposed by Everett Rogers. It states that the main aspect of information system adoption is compatibility referring to users’ beliefs that the innovation is reliable with their experience, lifestyle, existing values, and requirements. A high level of compatibility may lead to the better adoption of innovation. This describes the speed and pattern of how new ideas are promoted in a population and understand their behavioral strategy. The method by which innovations are spread in the population and the concept associated with innovative technology are important features of how fast the diffusion happens. In the marketing field, the diffusion theory is used for understanding and promoting the adoption of new services or products. Influencer marketing also uses the diffusion theory of innovation. For example, brands contact social media influencers to promote their services, use new products, and promote and become early adopters. It can be applied in health care to encourage patients to adopt advanced healthy technologies. The people involved in the diffusion theory are early adopters, innovators, initial majority, late majority, and slowcoaches [9,10].

Various advanced technologies like mobile shopping, e-commerce World Wide Web, internet banking, and mobile banking, the Internet, assistive social robots, e-learning adoption, digital payments, online transactions, online gaming, engineering, computer science, management, operation research and psychology have evaluated the TAM and DOI theory. TAM and DOI are two important outlines to understand how to adopt new technology. TAM works on two perceptions; usefulness and ease of use, While DOI explains how innovations are promoted in population encountering the aspects like compatibility and relative advantage. Both TAM and DOI provide a framework to analyze user attitude, design accessible technologies and make effectual approaches for successfully advertising innovative technologies. However, these have few limitations, so should be combined with other adoption theories to understand user financial controlling behaviors and discover more advancements [11]. In order to comprehend the acceptance of Apple Pay, current study aims to utilize the Diffusion of Innovation (DOI) and Technology Acceptance Model (TAM) theories. It stresses the significance of trust, security, lifestyle congruence, financial control, and relative advantage. TAM provides valuable insights into consumers’ behavioral intentions about digital payments, influencing their uptake of Apple Pay by emphasizing perceived effectiveness and simplicity of use. DOI’s focus on compatibility and innovation adoption ensures Apple Pay aligns with consumers’ values and lifestyles, resulting in a higher adoption rate. TAM and DOI collaborate to study consumer intentions towards Apple Pay, focusing on lifestyle congruence, security, and trust.

## 3. Literature review

The emergence of digital payment methods like Apple Pay has provided an easy and secure payment method for customers. Due to the progressing nature of digital payments, researchers are more focused on improving it. Although previous studies have extended m-banking research through a variety of theoretical frameworks and methodological approaches, there is still a shortage of sufficient data about the ongoing use of financial services after their initial adoption. Researchers have checked various theories and models to determine the influential aspects of digital payment services and they are more relying on the approval of Information systems, and implementation theories like TPB (theory of planned behavior), TAM (technology acceptance model), DOI (diffusion of innovation theory), as well as UTAUT that stands for Unified Theory of Acceptance and Use of Technology [4].

### 3.1 Fintech and digital payments

Fintech stands for financial technology that is used for improving digital payments. Fintech is used to help business owners, companies, and customers in managing their financial operations [12]. It is designed by specialized algorithms and software that works on smartphones and computers. In April 2018, SAMA introduced Fintech Saudi, a project that aims to accelerate Fintech development and diversify the financial sector. Under Fintech Saudi, several significant projects were introduced, such as the Fintech Access Guide, Fintech Saudi Podcast, Fintech Saudi Tour 19, Fintech Accelerator program, and Fintech Internship [13]. In recent years, sixteen Fintech companies have been granted licenses to offer digital insurance brokerage, consumer microfinance, and payment services. Among the first digital wallet businesses to receive SAMA’s license under the experimental permission scheme were Halalah (now called Hala) and Bayan Payments. Additionally, SAMA and Ripple have agreed on a deal to support Saudi banks with blockchain-based transaction settlement. Additionally, the number of Fintech start-ups in the Kingdom tripled, from twenty to sixty in the 2019–2020 fiscal year, according to the second edition of Fintech Saudi’s Annual Report, demonstrating the sector’s accelerating rate of development [14,15].

### 3.2 M-Wallets

A m-wallet is a virtual wallet on mobile phones that holds data and information from loyalty cards, credit or debit cards and coupons [16]. A mobile wallet is a virtual wallet that holds data from loyalty cards, credit or debit cards, and coupons [17]. With mobile wallets, consumers may pay in-store without carrying cash or actual credit cards. PayPal, Apple Pay, Samsung Pay, and Google Pay are some of the most widely used mobile wallets [18]. Apple Pay is one of the most famous digital payment applications in the world and also in KSA (Kingdom of Saudi Arabia) due to a massive number of iPhone users. Most people in KSA have an Apple device like iPad, MacBook, Apple Watch, or iPhone. This application can be installed from the app store on any Apple device. People are using Apple Pay to make purchases in apps and web- stores. Most sellers in Saudi Arabia receive payments via Apple Pay in stores [11,13]. Mobile wallets (m-wallets) contribute to environmental sustainability by reducing reliance on physical cash, paper receipts, and plastic cards, thereby minimizing waste and resource consumption. Digital transactions lower the carbon footprint associated with the production, transportation, and disposal of traditional payment methods [19,20]. However, the environmental impact of digital payments depends on factors like data center energy consumption and electronic device production, which require proactive privacy and security measures for widespread adoption [21,22].

### 3.3 Hypotheses development and conceptual framework

#### 3.3.1 The impacts of Perceived Financial Control (PFC) on Intention to Use Apple Pay (IU).

Perceived Financial Control focuses on financial matters. It refers to an individual’s belief in their ability to manage and control their financial resources, make effective financial decisions, and influence financial outcomes in their life. Financial matters may be of cost or time [23]. Apple Pay offers a regular payment system that saves customers from repeat or erroneous purchases. Additionally, you may get and redeem points when you use Apple Pay to make payments by using contactless card rewards in a wallet [24]. Mokhtar et al. [14] investigated customer loyalty in the context of mobile banking adoption and found that Ubiquitous Finance Control significantly impacts the adoption of mobile banking. A recent study by [12] carried out in the context of Bangladesh confirmed the significant impacts of PFC on the intention to use m-wallets. Hence, this study hypothesize:

**H1:** Perceived Financial Control significantly influences Intention to Use Apple Pay

#### 3.3.2 The impacts of Relative Advantage (RA) and Intention to Use Apple Pay (IU).

Relative advantage means perceived benefits to adopt a new technology as compared to other technologies. When comparing Apple Pay with other digital payments, it is easy to use, secure, and accessible. Apple Pay allows customers to pay with full confidence as it doesn’t monitor customer details or purchases. It can be accessed from anywhere at any time even if you don’t have an internet connection. You can make payments from Apple Watch without having a mobile phone [24]. Recent studies about m-wallet adoption and unified payment interface have confirmed that relative advantage significantly impacts the intention to use m-wallets and digital payments [12,25]. Hence, this study hypothesize:

**H2:** Relative Advantage significantly influences Intention to Use Apple Pay

#### 3.3.3 The impacts of Perceived Security (PS) on Intention to Use Apple Pay (IU).

Perceived security plays a crucial role in shaping users’ intention to adopt m-wallets [26]. The platform utilizes Near-Field Communication (NFC) technology for seamless interactions with payment terminals [27]. When a user’s phone is in close proximity to a terminal, Apple Pay automatically presents the default payment card, requiring authentication via Touch ID or Face ID before completing the transaction [28]. This multi-layered security mechanism ensures secure and efficient transactions, reducing concerns about fraud and unauthorized use. Hidayat-Ur-Rehman et al. [29] confirmed that perceived security significantly impacts m-wallet adoption, reinforcing its importance in digital payment behavior. Therefore, this study hypothesizes:

**H3a:** Perceived Security significantly influences Intention to Use Apple Pay

#### 3.3.4 The impacts of Perceived Security (PS) on Perceived Financial Control (PFC).

Perceived security and perceived financial control are two ideas that interrelate with each other [12]. Modern digital wallets like Apple Pay use secure technologies to protect user data and enable fast, cashless, and seamless financial transactions [30]. Apple collaborated with big banks and credit card organizations to provide their users with a seamless experience [31]. In their study on the role of consumers’ perceived security, perceived control, interface design features, and conscientiousness in the continuous use of mobile payment services, Zhang et al. [32] confirmed a significant relationship between perceived security and perceived control. Perceived Security (PS) significantly influences Perceived Financial Control (PFC) in m-wallets like Apple Pay adoption by enhancing users’ confidence in transaction safety, thereby increasing their perceived ability to manage finances effectively [12]. Therefore, this study hypothesizes:

**H3b:** Perceived Security significantly influences Perceived Financial Control

#### 3.3.5 The impacts of Perceived Security (PS) on Perceived Trust (PT).

Perceived security and trust are important concepts in adopting Apple Pay for financial management [3]. Apple Pay offers a trusted platform to its users with high-security features [33]. Apple Users trust that their data is safe on Apple Pay and can be only accessed using a touch ID or face ID. Perceived Security (PS) significantly impacts Perceived Trust (PT) by ensuring that users feel confident their transactions and personal information are protected [33]. This heightened sense of security builds a foundation of trust, making users more likely to rely on and continuously use mobile payment services, thus enhancing overall user experience and satisfaction. These arguments are supported by the findings of the prior research [29,34]. Therefore, this study hypothesizes:

**H3c:** Perceived Security significantly influences Perceived Trust

#### 3.3.6 The impacts of Perceived Trust (PS) and Perceived Financial Control (PFC).

The adoption of Apple Pay is influenced by both perceived financial control (PFC) and perceived trust (PT). High Perceived trust makes consumers feel that Apple Pay manages their money wisely due to its reputation and user-friendly interface [12]. Because features like transaction tracking and biometric authentication provide users with a sense of control over their spending, this in turn promotes a sense of financial control [35]. However, it is clear that Apple consistently prioritizes customer trust and financial management. Although prior research does not provide direct evidence for the impact of PS on PFC, Mokhtar et al. [14] confirmed that financial control and trust significantly impact mobile banking adoption and customer satisfaction. Therefore, it can be hypothesized that PS also significantly impacts PFC, warranting further examination. Thus, this study hypothesizes:

**H4a:** Perceived Trust significantly influences Perceived Financial Control

#### 3.3.7 The impacts of Perceived Trust (PT) and Intention to Use Apple Pay (IU).

The intention of using Apple Pay is significantly influenced by perceived trust. Users are more inclined to think about utilizing m-wallets for financial transactions if they think it is trustworthy and works in their best interests [36]. This trust is derived from things like Apple’s well-known brand, transparent security approaches, and easy-to-use interface. The impacts of trust on IU have been confirmed by numerous prior studies [29,34]. Thus, this study hypothesizes:

**H4b:** Perceived Trust significantly influences Intention to Use Apple Pay

#### 3.3.8 The impacts of Lifestyle Congruence (LC) and Intention to Use Apple Pay (IU).

Lifestyle Congruence refers to encompasses not only the practical fit but also the psychological and emotional alignment between the user’s self-concept, values, and aspirations and the perceived image or values associated with the product, service, or technology [37,38]. Apple Pay is adopted by people with fast and technology-oriented mindsets as they value security and privacy more than everything. Apple understands its audience and their lifestyle, so its services are congruent with them for wider adoption and efficient marketing. In research conducted by [39] on the effect of consumer-based brand equity and satisfaction on loyalty, they confirmed that lifestyle congruence influences customer satisfaction. Similarly, a study by [38] demonstrated that functional congruence significantly affects the behavioral intention to use smart wearable healthcare devices. In the context of this study, we propose significant impacts of lifestyle congruence on the intention to use Apple Pay. We believe that if users find Apple Pay congruent with their lifestyle, they will be more inclined to use it. Thus, this study hypothesizes:

**H5a:** Lifestyle Congruence significantly influences Intention to Use Apple Pay

#### 3.3.9 The impacts of Lifestyle Congruence (LC) on Relative Advantage (RA).

Relative advantage and lifestyle congruence both affect the use of Apple Pay. When compared with other options, Relative advantage highlights benefits like speed and ease that are attractive to people who like fast technologies [40]. On the other hand, Lifestyle Congruence indicates how well a product fits into a person’s lifestyle [41]. The intention of an individual to use Apple Pay may be obstructed by a low perceived RA, even if they believe Apple Pay to be somewhat in line with their lifestyle. Although prior research lacks evidence on lifestyle congruence impacting relative advantage, this study posits that if an innovation aligns with users’ lifestyles, it can positively influence their perceptions of its relative advantage, thereby enhancing their intention to use it. Thus, this study hypothesizes:

**H5b:** Lifestyle Congruence significantly influences Relative Advantage

#### 3.3.10 The impacts of Ease of Use (EOU) on Relative Advantage (RA).

Convenience, according to EOU, is the point at which a user believes they can utilize technology to finish their task easily, at a suitable time and location [42]. A service is only perfect if it saves its customers from emotional, physical, and cognitive burdens [43]. Apple Pay is a payment service that is secure, reliable, and convenient. Numerous studies have combined ease of use and relative advantage within the same model, confirming their significant impacts on the target variable. This study posits that if users find the innovation easy to use, they may also perceive it as more advantageous. Such results were confirmed by research in the context of m-wallets adoption [12]. A seamless user experience can enhance satisfaction and perceived value, reinforcing the belief that ease of use positively influences relative advantage. Based on this logic, this study hypothesizes:

**H6a:** Ease of Use significantly influences Relative Advantage

#### 3.3.11 Ease of Use (EOU) and Intention to Use Apple Pay (IU).

EOU relates to the degree to which the user believes that new technology is easy to use and useful in the sense of saving effort and time [44]. Apple Pay provides a user-friendly interface and seamless integration with Apple devices. Customers don’t need to carry their debit or credit cards as most stores allow them to make payments through Apple Pay. Many prior studies have confirmed the significant impact of Ease of Use (EOU) on Intention to Use (IU) [34,45,46]. Thus, this study hypothesizes:

**H6b:** Ease of Use significantly influences Intention to Use Apple Pay

**Fig 1:**
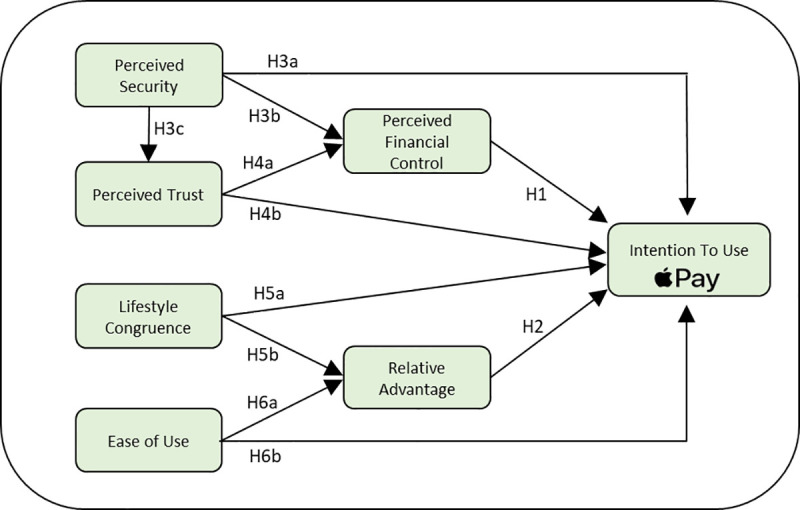
Proposed model of the study.

Proposed model of the study is depicted in Fig 1 below.

## 4. Research methodology

### 4.1 Scales development

This study employed a survey method to gather data for testing the proposed model. The survey comprised two sections: the first captured respondents’ demographic details, while the second included 28 items assessing key constructs related to Apple Pay adoption. To ensure participant relevance, a screening question confirmed that only Apple Pay users participated. The survey items were adapted from well-established scales in prior research on digital payments and Fintech. Specifically, perceived security and perceived trust were each measured using four items adapted from [47], while three items for lifestyle congruence were drawn from [48]. Additionally, four items for ease of use were adapted from [49], four items for perceived financial control from [14], and four items for relative advantage along with five items for intention to use Apple Pay from [50]. All items were measured using a 5-point Likert scale. The reliability and validity of the measurement instruments were rigorously assessed following the recommendations of [51]. Reliability was examined through internal consistency reliability, composite reliability, and indicator reliability. Convergent validity was assessed using the Average Variance Extracted (AVE). Discriminant validity was evaluated using the Fornell-Larcker criterion and the Heterotrait-Monotrait (HTMT) ratio. These assessments ensured the robustness of the measurement scales. Detailed results of the measurement model analysis are provided in Sections 5.1 to 5.5. The measurement items used in the study are provided under “Measurement Items in S1 Text.”

### 4.2 Data acquisition and sampling strategy

To test the proposed model, the researcher employed a combination of cluster sampling and purposive sampling. Cluster sampling was applied at the city level to ensure a geographically representative sample from Saudi Arabia’s administrative regions. The clustering was based on the 13 administrative regions defined by the [52], ensuring that major urban centers across different regions were included. Within each cluster, the capital city of each administrative region was selected, as capital cities typically have larger populations and stronger economic activity compared to other cities in the respective region. This selection ensures adequate representation of urban consumers, who are more likely to use digital payment systems like Apple Pay, given their better access to financial and technological infrastructure. The selected cities formed the sampling frame for the next stage of participant selection.

Within these pre-selected cities, purposive sampling was used to recruit respondents who actively use Apple Pay, as the study specifically investigates their adoption behavior. This non-probability sampling method was chosen because the objective of the study required data from individuals who have experience with Apple Pay transactions. Given the nature of the research, an online survey distribution strategy was adopted, leveraging social media platforms and digital payment-related forums to reach the target population efficiently. To ensure gender-based representation, the survey link was shared across diverse online platforms, including forums and groups with mixed-gender participation. However, as participation was voluntary, responses were received based on self-selection, without enforcing a strict gender quota. Institutional Review Board (IRB) approval was obtained before data collection, and participant recruitment took place over a three-week period, from December 14, 2023, to December 31, 2023.

The study employs partial least squares structural equation modeling (PLS-SEM) for data analysis. In determining an appropriate sample size, Roscoe [53] suggested an effective range between 30 and 500 participants, emphasizing the inaccuracy of very large samples. Stevens [54] recommended a minimum of 15 cases per predictor for multiple regression. Since SEM follows similar principles to multiple regression, a threshold of 15 cases per construct was deemed appropriate. A total of 237 responses were received. After screening for missing data and outliers, 16 cases were discarded, leaving 221 valid responses for analysis. The demographic distribution of respondents is presented in the Table 1 table below.

Although the selection of capital cities enhances geographic representation, the use of purposive sampling within these cities may introduce potential sampling bias. Specifically, the sample may over-represent urban, tech-savvy individuals who are active on digital platforms, while potentially underrepresenting users from smaller cities or those less engaged with online surveys. Additionally, since the study primarily recruited participants via social media and digital payment-related forums, individuals who are not frequent users of these platforms may have been under-sampled. Future research could address this limitation by incorporating a more diverse sampling strategy, including random sampling techniques or broader outreach to users in non-capital cities and offline settings. The complete dataset is available in the Supporting Information section as “S1 Dataset.”

**Table 1:** Demographics of the sample.

Category	Attributes	(%)
**Gender**	Male	53.8
	Female	46.2
**Age_Group**	18-25 years	39.4
	26-35 years	22.6
	36-45 years	19.9
	46-55 years	10.9
	More than 55 years	7.2
**Education**	High School	16.3
	Undergraduate	34.4
	Graduate	30.8
	Ph.D	18.6
**Employment**	Full Time	44.8
	Part Time	7.2
	Self-Employed	10.4
	Student	31.2
	Retired	6.3
**Apply_Pay usage Experience**	Below 6 months	7.2
	6-12 months	43.4
	1-2 years	31.7
	More than 2 years	17.6

## 5. Analysis of survey data

For data analysis, the statistical tools SmartPLS 4 and SPSS 23 were utilized, employing the PLS-SEM approach. Partial Least Squares Structural Equation Modeling (PLS-SEM) is a versatile and robust method for analyzing path models based on composites. It is particularly well-suited for exploratory research, complex frameworks, and studies with small to medium sample sizes [55,56]. Unlike Covariance-Based SEM (CB-SEM), which prioritizes model fit and theory validation, PLS-SEM emphasizes predictive accuracy and maximizing the explained variance of dependent variables [51]. This approach is especially beneficial for research involving multiple indicators and datasets with non-normal distributions.

One of the key advantages of PLS-SEM is its ability to function without assuming multivariate normality while efficiently managing complex moderating and mediating effects [57]. In this study, SPSS (v23.0) was used for preliminary data processing, including handling missing values, assessing common method bias (CMB), and checking linearity. Smart-PLS (v4.0) was then applied to test hypotheses and analyze structural relationships.

PLS-SEM was chosen over CB-SEM due to its superior capacity for managing intricate models, handling non-normal data, and prioritizing predictive power over model fit. Given the study’s focus on moderating and mediating relationships, PLS-SEM provides a more flexible and effective framework for evaluating direct and indirect effects. The data analysis process began with the assessment of the measurement model, followed by the analysis of the structural model. Detailed descriptions of the data analysis procedures are provided in Sections 5.1 and 5.2 below.

### 5.1 Common Method Bias (CMB)

Common Method Bias (CMB) occurs when data for both independent and dependent variables are gathered from the same source, such as a survey, potentially leading to systematic measurement errors [58]. To determine whether CMB was present in this study, Harman’s single-factor test was applied. The results revealed that a single factor explained 33.4% of the total variance, which is below the 50% threshold, indicating that CMB is not a concern. Additionally, a full collinearity test was conducted as an extra measure to assess the presence of CMB. The Variance Inflation Factor (VIF) values for all latent constructs were found to be below the recommended limit of 3.3, as suggested by [59]. This further confirmed that CMB does not pose a threat to the validity of the data. These findings reinforce the reliability of the study by ensuring that common method variance does not significantly influence the results.

### 5.2 Evaluation of the measurement model

To assess the measurement model, the PLS-Algorithm was run, resulting in an R² value of 0.622 for the target variable, intention to use Apple Pay. This indicates that the model explains 62.2% of the variance in Apple Pay usage. According to [51], an R² value between 0.50 and 0.74 denotes moderate explanatory power, suggesting that the model has a moderate level of explanatory capability for the variance in intention to use Apple Pay. This finding is aligning with similar technology adoption studies. In prior Fintech adoption studies using TAM, DOI, or similar models, explained variance (R²) in usage intentions ranges from moderate to high. TAM-based models often show moderate explanatory power (40–50% of intention variance). For example, one TAM extension explained 50% of the variance in students’ mobile wallet intention [60]. In contrast, more comprehensive frameworks that integrate TAM/DOI with additional factors achieve higher R² (often 60–70%) [61,62]. Thus, the 62.2% variance explained in our study is relatively high, approaching the upper range found in prior research.

Following [51] guidelines, the reliability and validity of the measurement model were assessed. Tests for this included internal consistency reliability, composite reliability, indicator reliability, and both convergent and discriminant validity.

Internal consistency was assessed using Cronbach’s alpha, with a threshold value above 0.6. Composite reliability was evaluated with a threshold of 0.7, and indicator reliability was verified against a benchmark of 0.7. As shown in Table 2, both composite reliability and Cronbach’s alpha values exceeded these thresholds, confirming the model’s reliability. Indicator reliability was validated as all outer loadings were above 0.7. Convergent validity was confirmed with average variance extracted (AVE) scores, listed in the third column of Table 2, all exceeding the 0.5 threshold.

To assess discriminant validity, two tests were conducted: the Fornell-Larcker criterion (FLC) and the Heterotrait-Monotrait (HTMT) ratio. The HTMT results, presented in Table 3, were below 0.9, supporting discriminant validity [63]. Additionally, the FLC indicated that the diagonal elements, representing the square roots of the AVE, were higher than the off-diagonal elements, further confirming discriminant validity. Thus, both tests confirmed the discriminant validity of the scales. Overall, all tests in the first phase confirmed that the scales used are both reliable and valid.

**Table 2:** Reliability & convergent validity tests summary.

Constructs	α > 0.7	Composite Reliability	AVE	Items	Indicators’ reliability
		>0.7	>0.5		>=0.7
Ease of Use	0.955	0.963	0.882	EOU1 EOU2 EOU3 EOU4	0.951 0.897 0.944 0.965
Intention to Use Apple_Pay	0.951	0.952	0.837	IU1 IU2 IU3 IU4 IU5	0.927 0.903 0.881 0.945 0.916
Lifestyle Congruence	0.949	0.953	0.907	LC1 LC2 LC3	0.931 0.959 0.967
Perceived Financial Control	0.904	0.907	0.779	PFC1 PFC2 PFC3 PFC4	0.901 0.928 0.889 0.808
Perceived Security	0.900	0.921	0.770	PS1 PS2 PS3 PS4	0.903 0.794 0.900 0.909
Perceived Trust	0.905	0.910	0.778	PT1 PT2 PT3 PT4	0.902 0.903 0.863 0.858
Relative Advantage	0.861	0.865	0.705	RA1 RA2 RA3 RA4	0.836 0.882 0.829 0.809

**Table 3:** Heterotrait-Monotrait ratio (HTMT).

Contructs	EOU	IU	LC	PFC	PS	PT	RA
**EOU**							
**IU**	0.291						
**LC**	0.114	0.322					
**PFC**	0.171	0.613	0.202				
**PS**	0.042	0.413	0.061	0.349			
**PT**	0.305	0.674	0.224	0.509	0.237		
**RA**	0.063	0.642	0.107	0.522	0.228	0.550	

### 5.3 Model fit indices

The model fit of the structural equation model was evaluated using multiple indices. The standardized root mean square residual (SRMR) was 0.149 for the estimated model, exceeding the acceptable threshold of 0.08, suggesting some discrepancy between observed and model-implied correlations. The discrepancy function measures, d_ULS (9.037) and d_G (1.167), indicate deviations in covariance structure, with d_ULS notably higher than the ideal range. The chi-square value for the estimated model was 1148.032, higher than the saturated model’s 935.853, which may be influenced by sample size sensitivity. The normed fit index (NFI) for the estimated model was 0.811, slightly below the recommended 0.90 threshold, suggesting a moderate model fit. A holistic evaluation is necessary for meaningful model interpretation.

### 5.4 Structural model analysis

**Fig 2:**
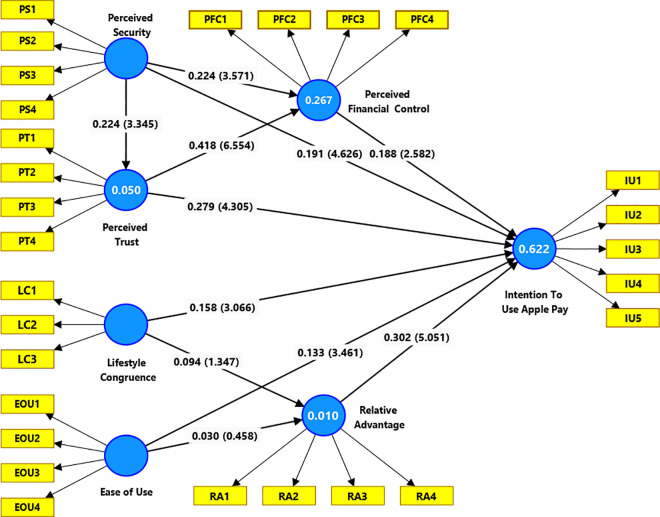
Bootstrapping results.

This research utilized a robust bootstrapping method with 10,000 subsamples and default settings to ensure accuracy and reliability. As shown in Table 4, the findings indicate that all proposed connections are statistically significant, except for the relationships between LC → RA and EOU → RA. Fig 2 illustrates the bootstrapping test results diagrammatically.

**Table 4:** Hypotheses testing results.

Hyp. #	Path	Path Coefficient	Standard Deviation	T Statistics	P Values	Remarks
**H1**	PFC → IU	0.188	0.073	2.582	0.010	Supported
**H2**	RA → IU	0.302	0.060	5.051	0.000	Supported
**H3a**	PS → IU	0.191	0.041	4.626	0.000	Supported
**H3b**	PS → PFC	0.224	0.063	3.571	0.000	Supported
**H3c**	PS → PT	0.224	0.067	3.345	0.001	Supported
**H4a**	PT → PFC	0.418	0.064	6.554	0.000	Supported
**H4b**	PT → IU	0.279	0.065	4.305	0.000	Supported
**H5a**	LC → IU	0.158	0.052	3.066	0.002	Supported
**H5b**	LC → RA	0.094	0.070	1.347	0.178	**Not Supported**
**H6a**	EOU → RA	0.030	0.065	0.458	0.647	**Not Supported**
**H6b**	EOU → IU	0.133	0.038	3.461	0.001	Supported

The first hypothesis proposed that Perceived Financial Control (PFC) significantly impacts the Intention to Use (IU) Apple Pay. This was supported by the statistical results (β = 0.188, t = 2.582, p = 0.010), indicating that stronger perceptions of financial control can lead users to adopt the payment service.

Regarding the influence of relative advantage (RA) on IU, the hypothesis was confirmed by the empirical results (β = 0.302, t = 5.051, p = 0.000). This implies that users are more likely to use Apple Pay if they perceive it as more advantageous than other services.

Exploring the role of perceived security (PS), the hypotheses proposed its significant impacts on IU (H3a), PFC (H3b), and perceived trust (PT) (H3c). These were supported by the results: H3a (β = 0.191, t = 4.626, p = 0.000), H3b (β = 0.224, t = 3.571, p = 0.000), and H3c (β = 0.224, t = 3.345, p = 0.001). These findings suggest that stronger perceptions of security enhance trust in the service, improve perceptions of financial control, and ultimately lead to the use of the service.

The proposed positive impacts of PT on both PFC (H4a) and IU (H4b) were also supported by the results: H4a (β = 0.418, t = 6.554, p = 0.000) and H4b (β = 0.279, t = 4.305, p = 0.000). These findings indicate that perceived trust plays a critical role in enhancing perceptions of financial control and positively affecting the intention to use.

In terms of Lifestyle Congruence (LC), it was found that if users perceive Apple Pay as aligning with their practical, psychological, and emotional needs, their intention to use the service is positively influenced (β = 0.158, t = 3.066, p = 0.002). However, the expected positive impacts of LC on RA were not supported (β = 0.094, t = 1.347, p = 0.178), indicating that perceptions of relative advantage are not influenced by the alignment of Apple Pay with practical, psychological, and emotional needs.

Lastly, the significance of ease of use (EOU) on both RA (H6a) and IU (H6b) was examined. While the ease of use did not influence perceptions of relative advantage (β = 0.03, t = 0.458, p = 0.647), it was found to positively affect the intention to use Apple Pay (β = 0.133, t = 3.461, p = 0.001). This suggests that a user-friendly service can attract more users to adopt Apple Pay, even if it doesn’t alter their perceptions of its relative advantage.

Table 5 extends the findings of Table 4 by analyzing the specific indirect effects, further validating the proposed model. The results indicate that several indirect relationships significantly contribute to the intention to use Apple Pay (IU). Specifically, the mediating role of perceived financial control (PFC) is confirmed, as seen in the significant indirect effect of perceived security (PS) on IU through PFC (β = 0.042, p = 0.033) and perceived trust (PT) on IU through PFC (β = 0.079, p = 0.023). Additionally, PS indirectly impacts PFC via PT (β = 0.094, p = 0.004), emphasizing the interconnected nature of trust and security in shaping user behavior. The indirect effect of PS on IU via PT (β = 0.063, p = 0.025) is also significant, reinforcing the role of perceived trust in bridging security perceptions and usage intention. Moreover, the multi-step path of PS → PT → PFC → IU (β = 0.018, p = 0.046) highlights a complex but significant influence pathway. However, the indirect paths involving ease of use (EOU) → relative advantage (RA) → IU (β = 0.009, p = 0.658) and lifestyle congruence (LC) → RA → IU (β = 0.028, p = 0.215) were found to be insignificant, indicating that neither construct plays a mediating role through relative advantage. These findings reinforce the primary direct effects observed in Table 4 and emphasize the importance of trust, security, and financial control in shaping user adoption.

**Table 5:** Hypotheses testing – specific indirect effects.

Path	Path Coefficient	Confidence Interval 95% Bias Corrected		T Statistics	P Values	Remarks
		LL	UL			
PS - > PFC - > IU	0.042	0.013	0.093	2.136	0.033	Significant
PS - > PT - > PFC	0.094	0.036	0.162	2.863	0.004	Significant
PT - > PFC - > IU	0.079	0.023	0.159	2.281	0.023	Significant
EOU - > RA - > IU	0.009	−0.028	0.053	0.442	0.658	Insignificant
LC - > RA - > IU	0.028	−0.007	0.084	1.241	0.215	Insignificant
PS - > PT - > IU	0.063	0.021	0.128	2.236	0.025	Significant
PS - > PT - > PFC - > IU	0.018	0.005	0.041	1.994	0.046	Significant

LL: Lower Limit; UL: Upper Limit; Significant; *p < 0.05.

This research highlights the significant factors influencing the intention to use Apple Pay, emphasizing perceived financial control, relative advantage, perceived security, trust, lifestyle congruence, and ease of use. These insights can guide strategies to enhance user adoption and satisfaction.

### 5.5 Multi-Group Analysis (MGA)

Our dataset comprises heterogeneous groups based on age and gender, which can influence the relationships between constructs. Ignoring this heterogeneity in PLS-SEM model evaluation may affect validity [64]. Researchers can address these differences using moderator analysis or multi-group analysis [65]. To examine the effects of these observable characteristics, the data was divided into two age-based groups: Older_Group (age > 35 years, 87 cases) and Younger_Group (age ≤ 35 years, 137 cases). Similarly, gender-based groups were formed: Male (119 cases) and Female (102 cases). Each group met the minimum sample size requirement. Since the highest number of arrows pointing to a latent variable in our model is eight, the minimum required sample size is 60 [64]. As all groups exceeded this threshold, sample size was not a limitation.

Moderation effects were assessed following [66] under two conditions: (1) Beta is significant in one group but not in the other, or (2) Both Betas are significant but have opposite signs. These criteria ensured a robust examination of moderation effects. Table 6 displays the MGA results based on age-specific group characteristics, while Table 7 presents the MGA results for gender-specific group characteristics.

**Table 6:** Age-wise group specific characteristics.

Hyp.	β (O.G)	β (Y.G.)	STDEV (O.G.)	STDEV (Y.G.)	p value (O.G.)	p value (Y.G.)	Remarks
**PFC → IU**	0.068	0.155	0.107	0.092	0.527	0.091	No Moderation
**RA → IU**	0.428	0.366	0.128	0.084	0.001	0.000	No Moderation
**PS → IU**	0.167	0.158	0.086	0.053	**0.053**	**0.003**	**Moderation**
**PS → PFC**	0.296	0.282	0.117	0.086	0.011	0.001	No Moderation
**PS → PT**	0.225	0.222	0.106	0.088	0.034	0.011	No Moderation
**PT → PFC**	0.322	0.395	0.110	0.083	0.004	0.000	No Moderation
**PT → IU**	0.232	0.228	0.107	0.082	0.031	0.005	No Moderation
**LC → IU**	0.125	0.155	0.088	0.067	**0.158**	**0.021**	**Moderation**
**LC → RA**	0.274	0.325	0.124	0.089	0.026	0.000	No Moderation
**EOU → RA**	0.027	−0.008	0.116	0.085	0.815	0.923	No Moderation
**EOU → IU**	0.174	0.175	0.069	0.050	0.012	0.000	No Moderation

**O.G.: Older_Group, Y.G.: Younger_Group.**

**Table 7:** Gender-wise group specific characteristics.

Hyp.	β (Female)	β (Male)	STDEV (Female)	STDEV (Male)	p value (Female)	p value (Male)	Remarks
**PFC → IU**	0.230	0.138	0.106	0.097	**0.029**	**0.157**	**Moderation**
**RA → IU**	0.242	0.347	0.087	0.078	0.005	0.000	No Moderation
**PS → IU**	0.177	0.207	0.048	0.070	0.000	0.003	No Moderation
**PS → PFC**	0.166	0.297	0.077	0.101	0.031	0.003	No Moderation
**PS → PT**	0.143	0.274	0.098	0.091	**0.145**	**0.003**	**Moderation**
**PT → PFC**	0.452	0.377	0.086	0.099	0.000	0.000	No Moderation
**PT → IU**	0.156	0.299	0.091	0.088	0.084	0.001	**Moderation**
**LC → IU**	0.338	0.126	0.110	0.069	**0.002**	**0.068**	**Moderation**
**LC → RA**	0.349	−0.113	0.114	0.101	0.002	0.267	**Moderation**
**EOU → RA**	−0.026	0.009	0.079	0.099	0.746	0.928	No Moderation
**EOU → IU**	0.128	0.132	0.044	0.058	0.004	0.024	No Moderation

#### 5.5.1 Age-wise group specific characteristics.

As indicated by the results in Table 6, moderation effects were observed in two out of the eleven hypothesized paths regarding age.

**PS → IU (Perceived Security → Intention to Use Apple Pay):** The Beta for the Older Group (0.167, p = 0.053) is positive but marginally insignificant, while for the Younger Group (0.158, p = 0.003), it is also positive and highly significant. This demonstrates a moderation effect, as the relationship between perceived security and intention to use Apple Pay is stronger and more significant for younger users. The significant effect in the younger group suggests that they are more influenced by security perceptions when adopting Apple Pay, while for the older group, security plays a relatively weaker role. This finding indicates that younger users, being more technology-oriented and aware of potential threats, prioritize security considerations more strongly when deciding to adopt Apple Pay, whereas older users may rely on other factors in their adoption decisions.

**LC → IU (Lifestyle Congruence → Intention to Use Apple Pay):** The Beta for the Older Group (0.125, p = 0.158) is positive but insignificant, whereas for the Younger Group (0.155, p = 0.021), it is positive and significant. This supports a moderation effect, indicating that lifestyle congruence is more relevant for younger users in shaping their intention to use Apple Pay. This supports a moderation effect, indicating that lifestyle congruence is more relevant for younger users in shaping their intention to use Apple Pay, as they are more inclined toward digital payment solutions that seamlessly integrate with their tech-savvy, fast-paced, and convenience-driven lifestyles. Their higher engagement with mobile technology and preference for digital financial tools make lifestyle congruence a crucial factor in their adoption decisions. The non-significant result for the older group suggests that their adoption decisions may be driven by other factors beyond lifestyle alignment.

Both paths confirm that age moderates the relationships, with younger users being more responsive to perceived security and lifestyle congruence when deciding to use Apple Pay.

#### 5.5.2 Gender-wise group specific characteristics.

Based on the results in Table 7, moderation effects were observed in five out of the eleven hypothesized paths regarding gender. Below is the revised explanation of each moderated path, incorporating the correct variable definitions:

**PFC → IU (Perceived Financial Control → Intention to Use Apple Pay):** The Beta for Females (0.230, p = 0.029) is positive and significant, while for Males (0.138, p = 0.157), it is positive but insignificant. This suggests a moderation effect, as the relationship between perceived financial control and intention to use Apple Pay is stronger and significant for females but not for males. This finding implies that female users rely more on their perceived financial control—such as their ability to manage finances effectively—when deciding to adopt Apple Pay. In contrast, male users may consider other factors, such as trust or perceived security, when forming their intention to use Apple Pay.

**PS → PT (Perceived Security → Perceived Trust):** The Beta for Females (0.143, p = 0.145) is positive but insignificant, while for Males (0.274, p = 0.003), it is positive and highly significant. This demonstrates a moderation effect, as the relationship between perceived security and perceived trust is stronger for males than for females. This suggests that male users place a higher emphasis on security concerns when forming trust in Apple Pay, whereas female users may consider other factors, such as ease of use or financial control, in building trust in the system.

**PT → IU (Perceived Trust → Intention to Use Apple Pay):** The Beta for Females (0.156, p = 0.084) is positive but marginally insignificant, whereas for Males (0.299, p = 0.001), it is positive and highly significant. This supports a moderation effect, indicating that trust plays a stronger role in influencing the intention to use Apple Pay among males compared to females. This finding suggests that male users are more likely to adopt Apple Pay if they trust the system, while female users might weigh additional factors, such as perceived financial control or lifestyle congruence, in their adoption decisions.

**LC → IU (Lifestyle Congruence → Intention to Use Apple Pay):** The Beta for Females (0.338, p = 0.002) is positive and highly significant, while for Males (0.126, p = 0.068), it is positive but only marginally significant. This confirms a moderation effect, as lifestyle congruence is a stronger predictor of intention to use Apple Pay for females than for males. This suggests that female users are more likely to adopt Apple Pay if it aligns with their lifestyle preferences, particularly if they perceive it as seamlessly integrating into their daily routines. Male users, on the other hand, may prioritize different aspects, such as security or trust, over lifestyle congruence in their decision-making process.

**LC → RA (Lifestyle Congruence → Relative Advantage):** The Beta for Females (0.349, p = 0.002) is positive and significant, while for Males (−0.113, p = 0.267), it is negative and insignificant. This demonstrates a moderation effect, as lifestyle congruence significantly predicts relative advantage for females but not for males. Female users who perceive Apple Pay as fitting well with their lifestyle are also more likely to perceive higher relative advantages, such as increased convenience, efficiency, and seamless integration with their financial habits. For male users, lifestyle congruence does not significantly impact their perception of relative advantage, suggesting they may base their adoption decision on other factors such as security or financial benefits.

The moderation effects indicate that gender plays a crucial role in shaping the relationships between key factors and Apple Pay adoption. Female users tend to be more influenced by perceived financial control, lifestyle congruence, and relative advantage, whereas male users emphasize perceived security and trust when deciding to use Apple Pay. These insights suggest that gender-specific marketing and user engagement strategies should be developed to cater to these differing concerns, ensuring that Apple Pay adoption strategies align with the unique preferences and motivations of both male and female users.

## 6. Discussion of the findings

This study highlights key determinants influencing users’ intentions to adopt Apple Pay, focusing on lifestyle congruence, security, trust, financial control, relative advantage, and ease of use. By integrating the Diffusion of Innovation (DOI) and Technology Acceptance Model (TAM) frameworks, the findings provide a comprehensive perspective on behavioral patterns driving mobile payment adoption. The results hold both theoretical and practical implications for the FinTech industry, offering insights into enhancing user confidence and engagement with digital payment solutions.

First, Perceived Financial Control (PFC) significantly influences Intention to Use (IU) Apple Pay, reinforcing the notion that users prefer payment solutions that provide financial autonomy. Users’ confidence in managing finances via Apple Pay likely stems from its features, such as real-time tracking and spending transparency [14]. Recent findings further confirm that digital payment systems enhancing financial control significantly boost adoption, as they enable users to monitor spending efficiently [67].

The study also underscores the impact of Relative Advantage (RA) on IU, suggesting that users prefer Apple Pay when they perceive it as superior to other payment methods. Features such as speed, security, and seamless integration with Apple services drive its perceived superiority, reinforcing the importance of highlighting these advantages in marketing strategies. This aligns with prior research indicating that ease of use and perceived benefits are critical motivators in mobile wallet adoption, as users favor platforms that streamline transactions and reduce cognitive effort [29,68].

Perceived Security (PS) plays a critical role in influencing IU, PFC, and Perceived Trust (PT), highlighting the necessity of robust security measures in financial technology adoption. Apple Pay’s biometric authentication and tokenization contribute to user confidence, reducing concerns about financial data security. By fostering trust and reinforcing users’ perception of financial control, enhanced security mechanisms positively impact adoption [29,32,34]. This finding aligns with recent studies indicating that heightened security measures in digital payments significantly reduce perceived risks, increasing adoption likelihood [69,70].

Perceived Trust (PT) emerges as a pivotal factor affecting both PFC and IU. Trust is essential in mitigating perceived risks and uncertainties associated with technology adoption. The strong PT-IU relationship suggests that trust-building efforts, including transparent policies, reliable customer support, and consistent performance, are crucial for increasing adoption. Additionally, the influence of PT on PFC indicates that trust enhances users’ confidence in managing their finances through Apple Pay. These outcomes are in agreement with those reported by earlier studies by [14,29,34]. These findings align with previous research demonstrating trust as a mediator between security perceptions and adoption intentions, fostering stronger financial engagement with mobile wallets [68,70].

Lifestyle Congruence (LC) significantly influences IU, indicating that users are more likely to adopt Apple Pay if it aligns with their psychological, emotional, and practical needs [38,39]. However, LC does not significantly impact RA, suggesting that while users appreciate the convenience of Apple Pay, it does not necessarily redefine their perception of its superiority over other payment methods. This finding underscores the importance of continuous innovation to emphasize unique benefits [29,67].

The study also reveals a dual role for Ease of Use (EOU) in influencing IU but not RA. A user-friendly interface encourages adoption but does not necessarily enhance perceptions of Apple Pay’s competitive advantage. This aligns with previous findings, which indicate that while ease of use facilitates initial adoption, other factors, such as perceived usefulness and financial benefits, play a more dominant role in shaping long-term perceptions [45,46]. Recent research further supports this conclusion, emphasizing that seamless integration with digital services and financial incentives drive perceived advantages more effectively than usability alone [68,71].

The multi-group analysis reveals that age and gender significantly influence the adoption of Apple Pay. Younger users are more responsive to security concerns, reflecting their heightened awareness of digital risks and data privacy. Their adoption decisions are also shaped by lifestyle congruence, as they prefer payment solutions that seamlessly integrate with their fast-paced, technology-driven routines. This suggests that digital payment providers should emphasize security features and convenience to attract younger consumers. Gender differences further highlight distinct adoption patterns. Female users are more likely to adopt Apple Pay when they perceive greater financial control, reinforcing the importance of transparency and expense management. Additionally, lifestyle congruence plays a more significant role for females, suggesting that personalization and seamless integration with daily routines enhance their adoption decisions. In contrast, male users prioritize trust and security, indicating that confidence in the platform’s ability to safeguard financial information is crucial for their acceptance of the service. These findings emphasize the need for targeted strategies to drive adoption. Marketing efforts should focus on security and trust for male users, while financial empowerment and lifestyle integration should be highlighted for female users. Understanding these variations can help financial technology providers tailor their services to meet the diverse needs of users effectively.

In conclusion, the empirical findings provide a comprehensive understanding of the factors driving the intention to use Apple Pay. By addressing users’ need for financial control, highlighting relative advantages, ensuring robust security, building trust, aligning with users’ lifestyles, and maintaining ease of use, Apple Pay can effectively enhance its adoption rates. The multi-group analysis further highlights that younger users prioritize security and lifestyle fit, while gender differences reveal that females value financial control and lifestyle congruence, whereas males emphasize trust and security. These insights suggest that Fintech providers should develop targeted strategies that cater to the distinct preferences of different demographic groups, ensuring a more personalized and effective approach to increasing adoption. Understanding these variations can help Apple Pay refine its user experience and marketing efforts to appeal to diverse consumer segments.

## 7. Implications of the study

This study enhances the theoretical understanding of technology adoption, particularly in financial technologies like Apple Pay. By integrating Diffusion of Innovations (DOI) theory and the Technology Acceptance Model (TAM), it presents a more comprehensive framework for digital payment adoption. The confirmation of perceived financial control (PFC) as a key determinant highlights the need to incorporate psychological factors like user confidence and financial autonomy into adoption models. Expanding beyond TAM’s traditional focus on perceived usefulness and ease of use, the findings suggest that financial control should be a core component in future theoretical models of financial technology adoption.

Additionally, the study highlights the critical role of perceived security (PS) and perceived trust (PT) in shaping users’ adoption intentions, aligning with trust theory and extending its application to digital payment systems. The strong influence of PS on PT, PFC, and intention to use (IU) underscores the necessity of security perceptions in fostering trust and encouraging adoption. This insight is particularly relevant to digital payments, where concerns about data security and fraud play a decisive role in user behavior.

The study also introduces a new perspective on lifestyle congruence (LC) in influencing IU, even though it does not impact relative advantage (RA). This suggests that while the functional benefits of a technology are important, its compatibility with users’ lifestyles can also be a major driver of adoption. This theoretical contribution encourages future research to place greater emphasis on lifestyle-related factors when analyzing technology adoption behaviors.

The study highlights the dual role of ease of use (EOU) in digital payment adoption, showing that while it significantly influences intention to use (IU), it does not directly impact relative advantage (RA). This suggests that EOU is essential for initial adoption, but long-term engagement relies more on perceived benefits. Future research should distinguish between factors driving initial adoption and those sustaining continued use, allowing for a more refined understanding of user behavior.

From a practical perspective, these findings provide valuable insights for developers, marketers, and policymakers aiming to enhance Apple Pay adoption. Developers should prioritize features that improve perceived financial control (PFC), such as real-time tracking, expense management, and financial transparency tools. These enhancements can boost user confidence and encourage adoption.

Marketers should emphasize Apple Pay’s relative advantages, including convenience, speed, and seamless integration with Apple’s ecosystem. These aspects should be communicated effectively through marketing campaigns, user education initiatives, and real-life testimonials to reinforce the service’s superior benefits over traditional payment methods.

Security remains a critical concern for users, making it essential for developers to implement robust security measures, such as biometric authentication and tokenization. However, simply having security measures is not enough—transparent communication about these features can build trust and alleviate concerns, driving higher adoption rates.

Policymakers should focus on regulatory frameworks that mandate high security and transparency standards, ensuring a trusted digital payment environment. Additionally, public education initiatives on the security and benefits of digital payments can further encourage broader adoption.

Finally, targeted marketing strategies are essential. Younger users prioritize security and seamless integration, while females value financial control and personalization. Males emphasize trust and security, indicating that tailored promotional strategies can improve user adoption and retention. Continuous user feedback and iterative improvements in usability will further enhance satisfaction and engagement.

In conclusion, this study provides valuable insights into the adoption of financial technologies like Apple Pay by integrating DOI and TAM frameworks. It highlights the importance of perceived financial control, security, trust, lifestyle congruence, and ease of use in shaping user behavior. The findings offer practical recommendations for developers, marketers, and policymakers to enhance adoption through improved security, financial management tools, and targeted marketing strategies. Future research should further explore the role of psychological and lifestyle factors in digital payment adoption, distinguishing between initial adoption drivers and long-term engagement. These insights contribute to both theoretical advancements and practical applications in financial technology adoption.

## 8. Limitations and future research avenues

This study has some limitations that should be acknowledged. First, it was conducted within a specific geographical and cultural context, limiting the generalizability of the findings. Future research should explore digital payment adoption in diverse regions to assess cultural influences. Second, the study used a cross-sectional survey approach, providing a snapshot of user behavior at a single point in time. Longitudinal studies could offer deeper insights into how perceptions and adoption intentions evolve with continued use of Apple Pay.

Another limitation is the reliance on self-reported data, which may introduce biases such as social desirability and recall bias. Future studies could integrate objective usage data, such as transaction records, to provide a more accurate assessment of user behavior. Additionally, while the model tested in this study is robust, it does not account for all possible adoption factors. Future research could examine social influence, cost considerations, and individual differences in technology readiness to enhance the understanding of digital payment adoption.

Furthermore, while lifestyle congruence was found to be significant, its interaction with other adoption determinants warrants further study. Future research could investigate how specific lifestyle characteristics, such as spending habits or technology affinity, impact adoption intentions.

Finally, as financial technologies and user expectations evolve, ongoing research is needed to keep technology adoption models relevant. Future studies should explore emerging trends, new security measures, and evolving financial ecosystems to provide updated insights into digital payment adoption.

## Supporting information

S1 Text Measurement items. (DOCX)

S1 Dataset Apple Pay data set. (CSV)
