# Supplementary material for: Factors influencing intentions to use Apple Pay: A behavioral perspective
Source: PLoS One. 2025 Jul 17;20(7):e0327122. doi: 10.1371/journal.pone.0327122 (PMC12270173; doi:10.1371/journal.pone.0327122)
Supplement: S1 Text — (DOCX) [file pone.0327122.s001.docx]

**Measurement Items**

**Perceived Security** adopted from [1]

PS1: The apple pay offers a safe environment to send sensitive information through.

PS2: I believe the apple pay has security measures to send sensitive information through.

PS3: I believe the transaction details in the apple pay is protected.

PS4: I would feel totally safe to provide my credit/debit card to purchase products through the apple pay.

**Perceived Trust** adopted from [1]

PT1: I trust the apple pay.

PT2: I think the apple pay always provides accurate financial services.

PT3: I think the apple pay interests my mind.

PT4: I think the apple pay always provides secure financial services.

**Lifestyle Congruence** adopted from [2]

LC1: Apple pay is expected to be comfortable.

LC2: Apple pay is expected to be fashionable.

LC3: Apple pay is expected to be priced appropriately considering their quality.

**Ease of Use** adopted from [3]

EOU1: It is easy to become skilful at using apple pay.

EOU2: Interactions with apple pay are clear and understandable

EOU3: It is easy to follow all the steps to use apple pay.

EOU4: It is easy to interact apple pay.

**Perceived Financial Control** adapted from [4]

PFC1: Using apple pay, I have control over my finances independent of place.

PFC2: Using apple pay, I have control over my finances independent of time.

PFC3: Using apple pay, I have control over my finances while travelling.

PFC4: It is always possible for me to transact with apple pay.

**Relative Advantage** adopted from [5]

RA1: Apple pay has more advantages than internet or cash payment systems.

RA2: Apple pay is more convenient than internet or cash payment systems.

RA3: Apple pay is more efficient than internet or cash payment systems.

RA4: Apple pay is more effective than internet or cash payment systems.

**Intention-to-Use Apple Pay** adopted from [5]

IUAP1: I expect my use of apple pay to increase in the future.

IUAP2: I intend to use apple pay in the future.

IUAP3: If I have an opportunity, then I will use an apple pay.

IUAP4: I will always try to use an apple pay.

IUAP5: I plan to use apple pay frequently.

References

1. Matemba ED, Li G. Consumers’ willingness to adopt and use WeChat wallet: An empirical study in South Africa. Technol Soc. 2018;53:55–68.

2. Talukder MS, Sorwar G, Bao Y, Ahmed JU, Palash MAS. Predicting antecedents of wearable healthcare technology acceptance by elderly: A combined SEM-Neural Network approach. Technol Forecast Soc Change. 2020;150:1–13.

3. Liébana-Cabanillas F, Marinkovic V, Ramos de Luna I, Kalinic Z. Predicting the determinants of mobile payment acceptance: A hybrid SEM-neural network approach. Technol Forecast Soc Change. 2018;129:117–30.

4. Shamsul Anuar Mokhtar, Hamidon Katan IH-R. MOBILE BANKING ADOPTION: THE IMPACTS OF SOCIAL INFLUENCE, UBIQUITOUS FINANCE CONTROL AND PERCEIVED TRUST ON CUSTOMERS’ LOYALTY. Sci Int. 2017;29(4):829–36.

5. Kaur P, Dhir A, Bodhi R, Singh T, Almotairi M. Why do people use and recommend m-wallets? J Retail Consum Serv. 2020;56:102091.
